# New insights in the interpretation of array-CGH: autism spectrum disorder and positive family history for intellectual disability predict the detection of pathogenic variants

**DOI:** 10.1186/s13052-016-0246-7

**Published:** 2016-04-12

**Authors:** Gerarda Cappuccio, Francesco Vitiello, Alberto Casertano, Paolo Fontana, Rita Genesio, Dario Bruzzese, Virginia Maria Ginocchio, Angela Mormile, Lucio Nitsch, Generoso Andria, Daniela Melis

**Affiliations:** Department of Translational Medical Sciences, Section of Pediatrics, Federico II University, Via Sergio Pansini 5, 80131 Naples, Italy; Department of Molecular Medicine and Medical Biotechnology, Federico II University, Naples, Italy; Preventive Medical Sciences, Federico II University, Naples, Italy; Telethon Institute of Genetics and Medicine, Pozzuoli, Italy

**Keywords:** Intellectual disability, aCGH, Copy number variant (CNV), Pathogenic CNV, Multiple congenital anomalies

## Abstract

**Background:**

Array-CGH (aCGH) is presently used into routine clinical practice for diagnosis of patients with intellectual disability (ID), multiple congenital anomalies (MCA), and autism spectrum disorder (ASD). ACGH could detect small chromosomal imbalances, copy number variations (CNVs), and closely define their size and gene content. ACGH detects pathogenic imbalances in 14–20 % of patients with ID. The aims of this study were: to establish clinical clues potentially associated with pathogenic CNVs and to identify cytogenetic indicators to predict the pathogenicity of the variants of uncertain significance (VOUS) in a large cohort of paediatric patients.

**Methods:**

We enrolled 214 patients referred for either: ID, and/or ASD and/or MCA to genetic services at the Federico II University of Naples, Department of Translational Medicine. For each patient we collected clinical and imaging data. All the patients were tested with aCGH or as first-tier test or as part of a wider diagnostic work-up.

**Results:**

Pathologic data were detected in 65 individuals (30 %) and 46 CNVs revealed a known syndrome. The pathological CNVs were usually deletions showing the highest gene-dosage content. The positive family history for ID/ASD/MCA and ASD were good indicators for detecting pathological chromosomal rearrangements. Other clinical features as eyes anomalies, hearing loss, neurological signs, cutaneous dyscromia and endocrinological problems seem to be potential predictors of pathological CNVs. Among patients carrying VOUS we analyzed genetic features including CNVs size, presence of deletion or duplication, genic density, multiple CNVs, to clinical features. Higher gene density was found in patients affected by ID. This result suggest that higher gene content has more chances to include pathogenic gene involved and causing ID in these patients.

**Conclusion:**

Our study suggest the use of aCGH as first-tier test in patients with neurdevelopmental phenotypes. The inferred results have been used for building a flow-chart to be applied for children with ID.

**Electronic supplementary material:**

The online version of this article (doi:10.1186/s13052-016-0246-7) contains supplementary material, which is available to authorized users.

## Background

ID (intellectual disability) and ASD (autism spectrum disorder) are life-long conditions with deficits in cognitive functioning (IQ<70) and adaptive skills that affects 1–3 % of children worldwide [1]. Array-CGH (aCGH) offers a high diagnostic yield, ranging from 14–20 %, for individuals with unexplained ID, ASD or multiple congenital anomalies (MCA) [2–5]. Available evidence suggests a change in the diagnostic approach for children with neuropsychiatric disorders and/or congenital anomalies, indicating the aCGH as the first-tier cytogenetic diagnostic test [5]. In 2014 the SIGU (Italian Society of Human Genetics) suggested that ID, of all severity, and/or ASD and/or epilepsy, hypotonia, dysmorphisms, growth alteration, congenital malformations might be associated with pathogenic aCGH results.

The chromosomal imbalances detected by aCGH are defined copy number variations (CNVs) that are referred as: microdeletions and microduplications of clear clinical relevance or pathogenic, variants of uncertain significance (VOUS) and benign polymorphisms [6–8]. The advances in molecular methodology of aCGH technology, *along with its* broader application, facilitated the detection of novel pathogenic CNVs. The significance of many VOUS still remains uncertain causing serious problems in defining their contribution in patients affected by ID, MCA and ASD [9–13].

The aim of this study was twofold:

1. To determine phenotypic clues associated to pathogenic CNVs, outlining criteria for selecting patients to be studied with aCGH as first-line test.
2. To identify cytogenetic criteria of VOUS to be taken into account for their potentially pathogenicity.

These aims allow us to depict an integrative flow-chart applying for children with ID.

## Study design

We present a retrospective-prospective study including 214 patients. In order to establish which patients could mostly benefit of aCGH as first molecular test, we tried to individuate clinical and anamnestic clues that significantly correlate with pathologic aCGH data. For this purpose, all the subjects received a complete history recall and an accurate clinical and instrumental evaluation. All the anamnestic-clinical-investigation data, either as a single feature or in combination, were correlated to pathologic or negative aCGH results.

Our second aim was to identify cytogenetic elements that could predict the pathological role of VOUS. We characterized meticulously all the VOUS detected, recording: the size of the rearrangements, the presence of deletions, duplications or multiple rearrangements and the genic density. We correlated the cytogenetic data with the severity of the phenotype. A severe phenotype was defined on the presence of severe ID, ASD and multiple malformations.

On the basis of inferred results, we define a flow-chart applying for children with ID.

## Patients and methods

### Patients

We enrolled 214 patients (114 males and 100 females with an average age of 5.63 years, range 1.3–19 years), with or without variable degree of ID, and/or ASD and/or MCA. They had been referred and evaluated at the Clinical Genetic Unit of the Translational Medical Science Department, section of Pediatrics, of the University of Naples “Federico II” in a 10-year period (2002–2012): 91 patients were recruited (47 male and 44 female, age range 1.7–19 years, mean 5.1 years) from 2002–2006, and followed up, so belonged to a retrospective part of the study; 123 patients (69 male and 54 female, age range 8 month-11years, mean 6.16 years) were enrolled in the prospective part of the study from 2006–2012.

## Methods

### Clinical-anamnestic data

The diagnostic workup started with a meticulous family history recall, physical examination focused on the presence of minor (dysmorphology evaluation) and major anomalies, neurological exam and assessment of the behavioral phenotype as detailed in the Additional file 1*.*

### Instrumental evaluation

The diagnostic instrumental evaluation included: brain MRI, abdominal ultrasound, echocardiography, EEG and ABR. Not all the investigations were performed due to the lack of patient cooperation or parental consent. For patients who underwent brain MRI the association between brain structural anomalies and the presence of ID (moderate/severe), microcephaly, macrocephaly, hearing loss, EEG abnormalities was evaluated.

### Metabolic screening tests

A metabolic screening panel (plasma amino acids, acylcarnitine profile, urine organic acids, ammonia and lactic acid) was performed. Targeted metabolic tests (lysosomal enzymes, urine oligosaccharides, transferrin isoform profile, plasma sterol concentrations, congenital glycosylation defects) were carried out when clinical suspect arose.

### Cytogenetic analysis

For patients included in the retrospective part, initial tests to exclude genetic syndromes suspected based on the facial and gestalt phenotypes or due to initial unavailability of aCGH were performed, including: karyotype (standard and high resolution type), specific loci (22q11.2, 7q11.23, 16p13.3) and/or subtelomeric FISH, DNA methylation analysis for Prader-Willi and Angelmann syndrome and FRAX-A/E test. Since a diagnosis was not achieved through these procedures, aCGH with an average resolution of 500 Kb was performed. In patients enrolled in the prospective part of the study, aCGH with a resolution of 50–75 Kb was used as first tier test. Different platforms have been used along with progressive improvement of aCGH technology. Despite this could represent a bias we could not analyze again all the patients previously studied in the retrospective part with a lower resolution power. We re-analyzed only few patients with clinical features strongly suggestive for chromosomal disorder and showing normal results. These results have been considered in the prospective part of the study.

Cytogenetic techniques applied and CNVs interpretative processes are included in Additional file 1*.*

The CNVs size, the presence of multiple rearrangements and the number of genes located in were assessed. Patients with overlapping chromosomal rearrangements have been compared and studied.

### Statistical analysis

Clinical data were assessed using a standardized protocol (excel sheet) for collection of data in all children. SPSS software (version 20.0; SPSS Inc., Chicago, IL, USA) and R (version 2.5.0; The R Foundation for Statistical Computing) were used for the statistical analysis. Analyses included only available data and missing values were not imputed. Data were summarized as means ± standard deviation (median [25^th^–75^th^ percentile]) for continuous variables and as frequencies (%) for categorical variables. The association between the presence of a pathogenic CNV (as opposed to a normal array CGH results) with clinical characteristics (including ID, ASD, and familiarity with ID and with several anomalies/malformations) was based on a standard two-step approach. In particular, univariate association between each predictor and the aCGH results were first established using chi-square test (or the fisher exact test when appropriate); afterward, those factors showing a bivariate association with the dependent variable at a *p*<0.2 were entered in a multivariate logistic regression model using a backward stepwise method for the selection of variables. The discriminatory ability of the final model was assessed using ROC curve analysis. In order to correct the area under the ROC curve (AUC) for over-optimism, which occurs when the fit of a model is evaluated using the same data in which the model was built, a bootstrap procedure as described in [14] was used. The exact Mann–Whitney U test was used for comparing children with and without ID in case of VOUS with respect to the following variables: presence of deletions/duplications, number of multiple rearrangements, average size of rearrangements and genetic density.

## Results

### Clinical data

In our cohort 168/214 patients showed ID: mild, moderate and severe ID was identified in 129, 25 and 14 patients respectively. All the clinical findings recorded along with their frequency are shown in Table 1.

**Table 1:** Clinical features recorded in each patient expressed as percentage in are provided

Signs/symptoms		Percentage of patients
Intellectual disability	Severe	6.5 %
	Moderate	12 %
	Mild	60 %
	Absent	21.5 %
Autism spectrum disorder		17.2 %
Familiarity for ID and/or MCA		8 %
Prenatal perinatal problems		14 %
Short stature		19.15 %
Tall stature		2.8 %
Macrocephaly		7 %
Microcephaly (Craniosynostosis included)		31 %
Forehead and eyebrows phenotypic abnormalities		38 %
Eyes, palpebral fissures and eyelashes phenotypic abnormalities		54 %
Nose and phyltrum phenotypic abnormalities		37 %
Oral region, teeth and tongue phenotypic abnormalities		49 %
Ears phenotypic abnormalities		37 %
Neck and thorax phenotypic abnormalities		18 %
Upper limbs, lower limbs, hands and feet phenotypic abnormalies		26 %
Gastrointestinal malformations (megacolon, Duodenal/Esophageal stenosis)		16 %
Neurology (Epilepsy, hypertonia, hypotonia, paresis, extrapyramidal signs)		14 %
Cardiac malformations		33 %
Pulmonary malformations		1 %
Kidney and urinary tract anomalies		8 %
Abnormal external genitalia		12 %
Ocular malformation		16 %
Vertebral anomalies		22 %
Skeletal dysplasia		5 %
Haematological abnormalities		3 %
Nails and hair anomalies		13 %
Alterated skin pigmentation or skin hemangioma		13.3 %
Endocrinological abnormalities		10 %

### Instrumental evaluation

All the results from instrumental evaluation are presented in Table 2. Currently, a consensus on the role of neuroimages in patients with ID has not been achieved. Brain MRI findings have to be considered of contributory value but not essential in defining a genetic diagnosis or in the assessment of children with ID. Brain MRI was performed in 122 out 214 patients (57 %) and 57 of 122 (47 %) showed structural malformations in line with previous report [3]. To date consensus to perform brain MRI include macrocephaly, microcephaly, asymmetric neurologic signs, intractable epilepsy or focal seizures, abnormal movements, hypotonia or progressive neurological disorder [2, 15]. In our cohort we did not find any correlation between brain MRI pathological findings and microcephaly (*p*= 0.652) or macrocephaly (*p*=0.473). Conversely pathologic MRI findings were correlated with both moderate/severe ID (*p*=0.046) and neurosensitive hearing loss (*p*=0.01).

**Table 2:** Genetic and Instrumental tests performed in the cohort are indicated

Diagnostic tests	Percentage of patients testing pathologic	Percentage of patients performing the test
ABR	39 %	41 %
EEG	37 %	29 %
Brain MRI	47 %	57 %
Karyotype	0 %	71 %
FRAX-A	0 %	26 %
Prader-Willi/Angelman	0 %	6 %
FISH 22q11.2	0 %	6 %
FISH 7q11	0 %	2 %
Rubinstein-Taybi	0 %	6 %
Subtelomeric rearrangements analysis	0 %	6 %
Other molecular analyses	0 %	21 %
Plasma amino acids and acylcarnitines	0 %	59 %
Urine organic acids	0 %	46 %
Plasmatic ammonia and lactic acid	0 %	40 %
Oligosaccharides (urine)	0 %	13 %
Glycosaminoglycans (urine)	0 %	11 %
Serum transferrin isoform profiling	0 %	9 %
Sterols (plasma)	0 %	6 %

Percentages of positive and negative results are pointed out

### Metabolic profile

The metabolic screening panel consisting in: plasma amino acids, acylcarnitine profile, urine organic acids, ammonia and lactic acid were applied in around 50 % of patients. In specific cases lysosomal storage disease were partially ruled out performing lysosomal enzymes assay, urine oligosaccharides and glycosaminoglycans. Transferrin isoform profiling and sterols were performed only in 9 and 6 % of patients (Table 2).

### Cytogenetic analysis results

#### Detection rate

**Fig. 1:**
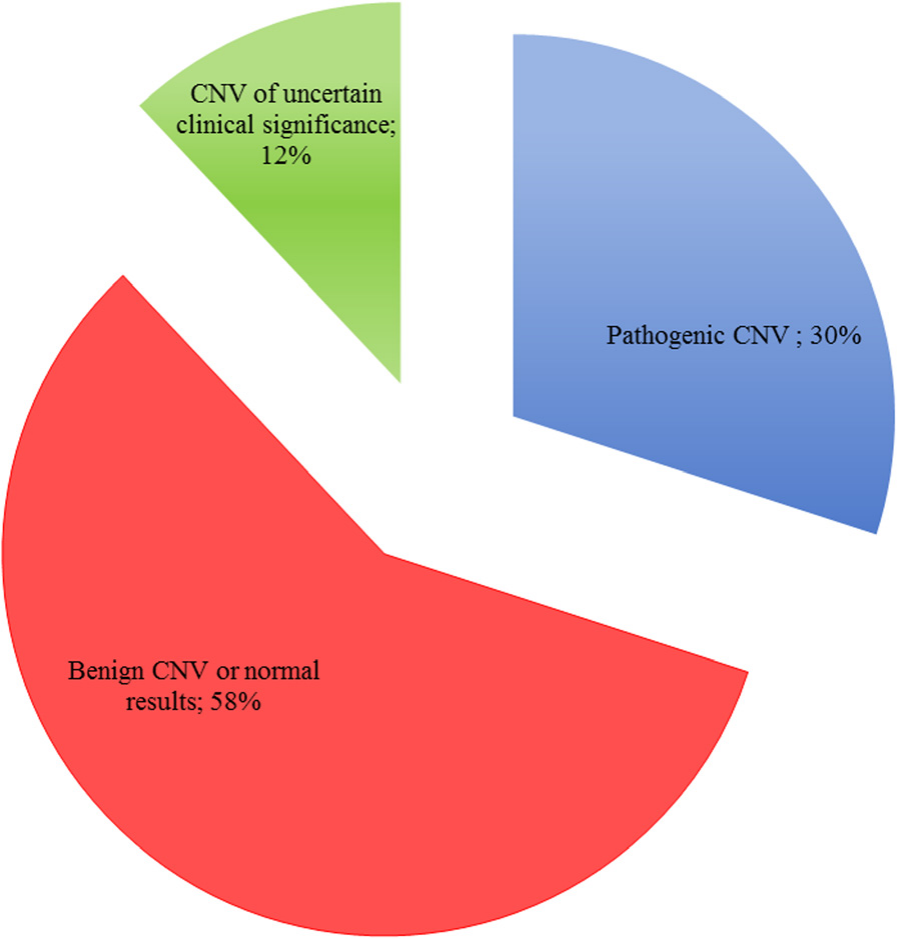
ACGH results are depicted

**Fig. 2:**
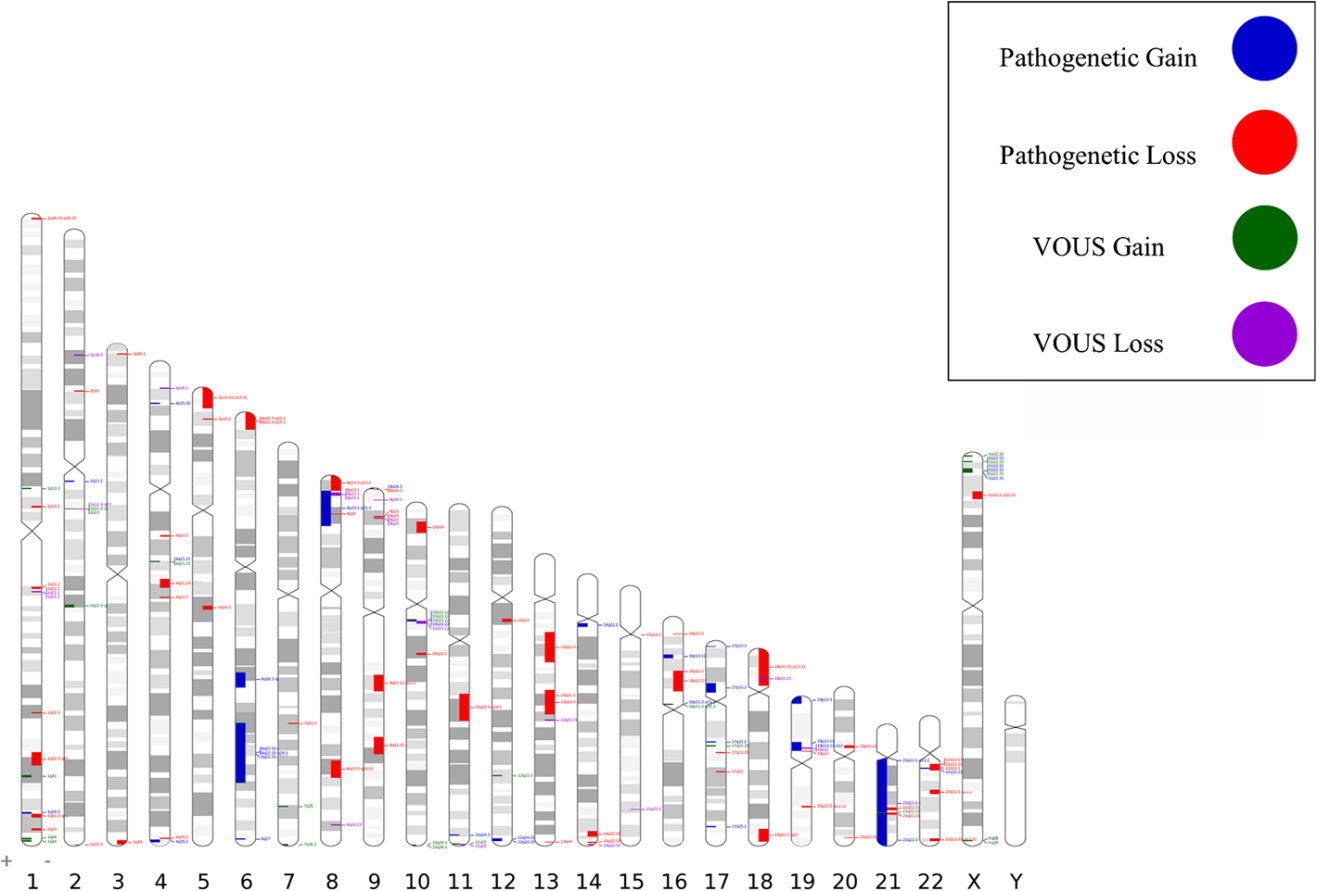
Overview of 90 chromosomal imbalances by aCGH (VOUS and pathogenic results included). See Legend for interpretation of markers

Pathological aCGH results was obtained in 65 patients (30 males and 35 females) (30 %), 124 patients (58 %) showed negative results or benign CNVs, while in 25 cases (12 %) VOUS were detected (Fig. 1). Figure 2 summarizes genetic loci of all detected pathogenic CNVs and VOUS. Pathological CNVs showed the highest average size of chromosomal rearrangement and gene content, a higher prevalence of deletions and of multiple rearrangements than those detected in VOUS and benign CNVs (Table 3).

**Table 3:** Cytogenetic features of detected CNVs

	Average size of chromosomal rearrangement (kb)	Deletions	Duplications	Gene Content	Multiple chromosomal rearrangements (number of patients)
VOUS (25)	523.30	13	13	2.73	9
Pathological CNV (65)	7018.2	65	42	34.67	29
Benign (26)	364.72	19	25	2.52	11

It should be noticed that pathogenic CNVs shared larger rearrangements, high number of deletions and multiple rearrangements

#### Specific syndromes

The diagnosis of already known genetic syndromes was achieved in 47 patients (data not shown). For Cri-du-chat syndrome, DiGeorge syndrome, 1q21.1 deletion syndrome, 1q21.2 deletion syndrome, 3q29 deletion syndrome, 6p25 deletion syndrome, 6q duplication syndrome, 13q12.3 deletion syndrome and 16p11.2 deletion syndrome, more than one case was recorded. The comparison of these cases might widen the phenotype associated to microdeletion syndromes and might contribute to a more careful study of critical regions containing haplosensitive genes. Furthermore, 18 pathogenic novel chromosomal rearrangements have been discovered as well.

#### Correlation study results

Table 4 shows the distribution of the clinical features and anomalies/malformations in children with pathogenic and negative aCGH results. In a univariate analysis, the only predictive factors of pathologic array results were: ASD and positive family history for ID/MCA/ASD. No significant differences in the frequencies of facial and non-facial dysmorphic features, congenital anomalies were recorded. ID, dysmorphic eyes anomalies, conductive and neurosensorial hearing loss, neurological signs (epilepsy, hypertonia, hypotonia, and paresis), cutaneous dyschromia and endocrinological problems were considered as potential predictors due to an association with the outcome at a liberal p value of 0.2. A multivariable logistic regression model with backward selection, showed that ID (OR 2.57; 95 % CI [1.07–6.2]; *p*=0.035), familiarity with ID/MCA/ASD (OR 11.4; 95 % CI [3.21–40.49]; *p*=<0.001), and cutaneous dyschromia (OR 2.75; 95 % CI [1.19–6.34]; *p*=0.018) were all independent predictors of a pathogenic CNV and yielded an optimism-corrected estimate of the overall accuracy, in terms of AUC, of 0.69 (the bootstrap estimate of the optimism in AUC was very small and equal to 0.01). The Chi-square Hosmer and Lemeshow were equal to 4.78 with a p value of 0.44. We enrolled 37 patients with ASD, in presence or absence of ID, 23 males and 14 females. Seventeen showed pathological CNVs and 4 VOUS. 12 patients carried out one or more microduplications, 9 microdeletions and 2 both microdeletion and microduplication.

**Table 4:** Correlations between single and combined clinical features and pathogenic and negative aCGH results are shown

Clinical signs, dysmorphic features	aCGH results (number)			
	Negative (115)	Pathological (60)	Overall (175)	*p*
Intellectual Disability	83 (72.2)	49 (81.7)	132 (75.4)	0.166
Autism Spectrum Disorders	13 (11.3)	14 (23.3)	27 (15.4)	0.037
Familiarity for ID/MCA/ASD	3 (2.6)	9 (15)	12 (6.9)	0.002
Prenatal perinatal problems	17 (14.8)	9 (15)	26 (14.9)	0.934
Short stature	21 (18.3)	12 (20)	33 (18.9)	0.781
Tall stature	3 (2.6)	1 (1.7)	4 (2.3)	1.000
Macrocephaly	8 (7)	4 (6.7)	12 (6.9)	1.000
Microcephaly (or craniosynostosis)	30 (26.1)	16 (26.7)	46 (26.3)	0.934
Forehead and Eyebrows dysmorphisms	41 (35.7)	21 (35)	62 (35.4)	0.932
Eyes, palpebral fissures and eyelashes dysmorphisms	53 (46.1)	36 (60)	89 (50.9)	0.081
Nose and philtrum dysmorphisms	40 (34.8)	23 (38.3)	63 (36)	0.642
Oral region, teeth and tongue dysmorphisms	45 (39.1)	28 (46.7)	73 (41.7)	0.337
Ears dysmorphisms	43 (37.4)	24 (40)	67 (38.3)	0.736
Neck and thorax anomalies	21 (18.26)	14 (23.73)	35 (20.11)	0.394
Upper and lower limbs, hands, feet dysmorphisms	24 (20.9)	12 (20)	36 (20.6)	0.893
Hearing Loss	15 (13)	13 (21.7)	28 (16)	0.140
Gastrointestinal malformations	15 (13)	5 (8.3)	20 (11.4)	0.353
Brain Malformations	13 (11.3)	8 (13.3)	21 (12)	0.695
Neurologic signs (Epilepsy, hypertonia, hypotonia, paresis)	15 (13)	4 (6.7)	19 (10.9)	0.198
Cardiac malformations	36 (31.3)	23 (38.3)	59 (33.7)	0.350
Pulmonary malformations	2 (1.7)	1 (1.7)	3 (1.7)	1.000
Kidney and urinary tract anomalies	3 (2.6)	1 (1.7)	4 (2.3)	1.000
Abnormal external genitalia	15 (13)	7 (11.7)	22 (12.6)	0.794
Eye malformations	12 (10.4)	7 (11.7)	19 (10.9)	0.804
Vertebral anomalies	29 (25.8)	14 (23)	43 (24.57)	0.718
Skeletal dysplasia	7 (6.1)	3 (5)	10 (5.7)	1.000
Haematological abnormalities	2 (1.7)	3 (5)	5 (2.9)	0.219
Nails and hair anomalies	16 (13.9)	6 (10.2)	22 (12.6)	0.482
Alterated skin pigmentation	8 (7)	9 (15)	17 (9.7)	0.088
Skin hemangioma	3 (2.6)	1 (1.7)	4 (2.3)	1.000
Endocrinological anomalies	9 (7.8)	9 (15)	18 (10.3)	0.138

Statistical significant correlations exist between pathologic CNVs and ASD and familiarity for ID/ASD/MCA. Other clinical features: ID (independent from severity), dysmorphisms of eyes, palpebral fissures and eyelashes, Hearing Loss, neurologic signs, abnormal skin pigmentation and endocrinological anomalies) appear to be potential predictors of pathological aCGH results

#### Characterization of uncertain CNV (VOUS)

Different clinical features (as single data or in combination) were correlated to cytogenetic features of *de novo* VOUS (CNVs dimension, presence of deletion vs duplication, genic density, presence of multiple different CNVs) (Table 5). We found that in children with ID, VOUS had a significantly higher gene density than in patients without ID (*p*=0.019). No associations were found between VOUS cytogenetic data and other analyzed clinical features (ASD, short stature, tall stature, macrocephaly, microcephaly, craniosynostosis, hearing loss, hepatic and gut malformation, brain malformation, epilepsy, heart malformation, genitourinary malformation, eye malformations, skeletal dysplasias, immunological anomalies, skin hyper/depigmentation, endocrinological problems). Moreover the severity of clinical phenotype did not correlate with any cytogenetic characteristics shared by VOUS.

**Table 5:** Evaluation of cytogenetic indicator in VOUS suggests that gene density is the only parameter associated to ID

	Intellectual disability			
	Absent (*n*=4)	Mild/Moderate (*n*=21)	Total (*n*=25)	*p*
Deletion; *n* (%)	3 (75 %)	10 (47.6 %)	13 (52 %)	0.593
CNV dimension; Median [25^th^ 75^th^ percentile]	367.5 [145.75;801.5]	258 [160.5;791]	258 [160.5;791]	0.902
Genic densitiy; Median [25^th^ 75^th^ percentile]	0.5 [0;1]	2 [1;4.25]	2 [1;3.25]	0.019
Multiple CNVs; Median [25^th^ 75^th^ percentile]	1 [1;2.5]	1 [1;2]	1 [1;2]	0.858

#### Suggested diagnostic flow-chart

**Fig. 3:**
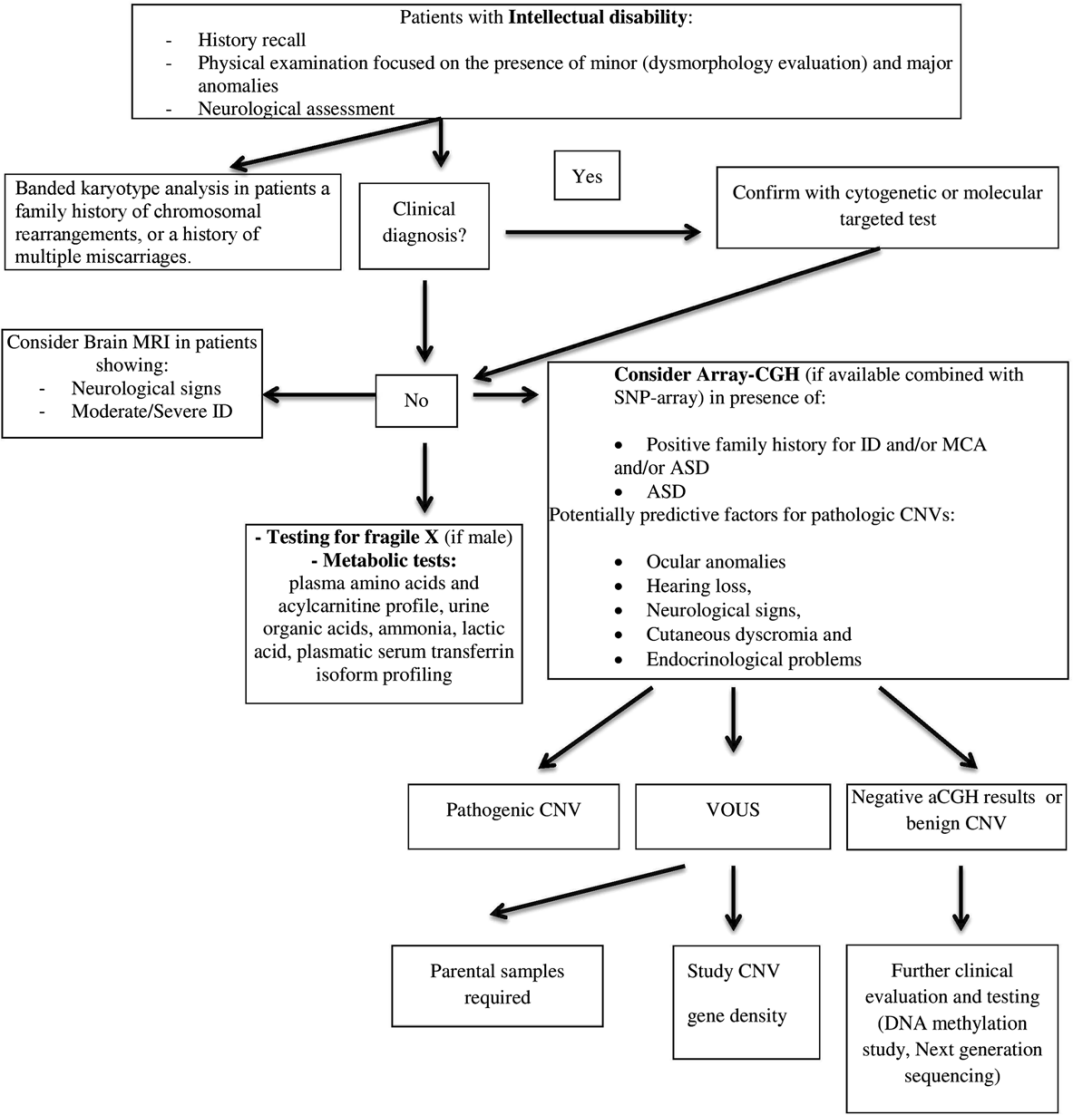
Algorithm in patients with unexplained ID and/or and/or ASD. After the collection of appropriate clinical and family history, you need to take a detailed physical and dysmorphology examination. If patient has a recognizable pattern of signs and symptoms you have to confirm diagnosis by cytogenetic or molecular targeted test. Nonetheless the infrequent detection rate Fragile X A/E syndrome should be excluded in all patients with ID. If the patient does not present with features of recognizable syndrome or metabolic disorder or the latter resulted negative for a suspected syndromes aCGH is the first-tier test especially in case of ASD diagnosis or family history positive for ID/MCA/ASD. Other potential predictors of pathogical results are: ocular anomalies, hearing loss, neurological signs, cutaneous dyscromia and endocrinological problems. If aCGH comes back negative further clinical investigations are warranted. If the detected CNV includes relevant region/genes or the gene content and its size meet guidelines criteria the result has to be considered pathogenic. In such cases parental studies and evaluation of cytogenetic feature as gene density could aid in ascertain their likely pathogenicity

Since ID/ASD/MCA are conditions of great concern and deserve special care, a flow-chart regarding the most appropriate care for patients with ID (including metabolic screening test, neuroimaging evaluation, diagnostic molecular investigations) has been depicted on the basis of the data inferred from this study (Fig. 3). We matched the data we obtained with the large-scale studies sharing a similar aim [16–19]. In the present work we could not use the combined technology “aCGH and single-nucleotide polymorphism (SNP) array”. We suggest to use this combination, when available, to detect CNVs plus a limited amount of SNP data to screen for absence of heterozygosity [19].

## Discussion

ID is a developmental disability, which presents in infancy or early childhood characterized by impaired intellectual functioning and adaptive behavior. The prevalence is estimated between 1 and 3 % of children [5, 20–22]. ASDs are clinically heterogeneous disorders that include: autism, Asperger syndrome, pervasive developmental disorders not otherwise specified, and childhood disintegrative disorder. ASD ha*s* been estimated to affect 1/100 to 1/150 children [3]. Many children with ASD also have ID representing together the most frequent referral to geneticists for a diagnostic workup. American Academy of Pediatrics Committee has discussed the importance of early identification of the cause of neurocognitive phenotype identifying several benefits [3]. Since even small chromosomal anomalies have been established as a major cause of ID and ASD, aCGH test has become an important diagnostic tool for patients at least sharing neuro-phenotypes [2–5].

Despite a very high number of studies describing genetic findings of CNVs, we identifed a high rate of pathologic CNVs, more frequently deletions, in a cohort of patients with ID and/or ASD. High gene content is also found in patients with VOUS and ID.

We also demonstrated that a positive family history for ID/ASD/MCA (cardiologic, renal, intestinal anomalies) and ASD were two good independent indicator of pathologic CNVs.

On the basis of our data we suggest that aCGH should be used as first tier diagnostic test in the presence of ID/ASD/MCA. We also underline the importance of family historical recall and of parents’ clinical observation in order to evaluate the presence of ID/ASD in the parents.

Among VOUS, higher gene density was found in patients affected by ID.

A diagnosis of metabolic disorders has been reported in 1–5 % of patients with ID [3, 19, 23, 24]. Diagnostic evaluation for inborn errors of metabolism (IEMs) was performed in 126 patients giving normal data. Notwithstanding, metabolic studies should be thoroughly considered in patients with ID due to the potential treatability of IEMs.

ACGH was performed in patients included in the retrospective part of this study because of negative results of other genetic studies, while it was applied as first tier test in the prospective part of the study because growing number of papers highlighted its diagnostic power [25–27]. We identified 65 (30 %) and 25 (12 %) patients with pathogenic CNVs and VOUS respectively. The detection rate of clinically significant CNVs is about 30 % therefore higher than the yields (from 14.2–21.1 %) obtained from studies that used similar platforms [2, 3, 5, 10, 28–30]. We speculate that the higher prevalence of pathological CNVs in our study is potentially ascribed to the careful selection of patients. Among patients with pathological CNVs, the deletions were more frequent than duplications (65 vs 42) being the former more commonly interpreted as pathogenic [10]. The pathogenic CNVs have a higher size (7 Mb) and gene density (35 genes) than VOUS and benign ones in agreement with other reports [29, 31, 32]. 29 patients with pathological CNVs have multiple rearrangements, which are known to exacerbate neuro-developmental phenotypes (Table 3) [5, 32]. In these patients parental chromosomal study did not reveal balanced translocations.

We diagnosed 47 patients (22 %) with OMIM syndromes on the basis of either overlapping described well- microdeletion/microduplication syndromes or known causing-genes mapped within chromosomal rearrangements. ACGH detected CNVs scattered throughout the genome, but the chromosome 1, 8, 22, X resulted most frequently involved in line with other reports [33]. The non-random involvement of specific chromosomal segments could be the results of non-allelic homologous recombinant mutational mechanism [29]. Other recurrent pathogenic CNVs involved 1q21.1, 1q41q42, 2p15, 16p13.1, 16p11.2, 17q21.31 allowing us to characterize the phenotypes associated to chromosomal rearrangements in these specific regions [30, 34–39]. A specific chromosomal abnormality does not always correspond to a specific or suggestive phenotype. In such cases, the detection of genomic aberration precedes the definition of specific phenotypes [30]. In our dataset patients with 22q11.21 deletion and Cri-du-chat syndromes showed an atypical phenotype making the clinical diagnosis challenging [40].

The potential limitations of aCGH application regard: delayed turnaround time, the impossibility of the detection of balanced translocations and low-level mosaicisms, the high costs, so that clinical criteria for selection of patients with higher probability of pathogenic CNV are desirable.

The selection of patients who are most likely to have a diagnosis by aCGH, minimizing the number of benign CNVs or negative results, remains an attractive goal [41]. This study represents the largest collection of specific clinical and instrumental data for which an association with pathologic CNVs has been investigated. From previous studies, the same rate of pathologic chromosomal imbalances by aCGH was found in unselected and selected patients ([18, 41] respectively). Other studies found the higher frequency of pathogenic CNVs in patients with congenital anomalies, unspecified dysmorphisms, growth anomalies, heart defects, primary microcephaly and familial occurrence of ID [22, 42–44]. The diagnostic yield among patients with more severe ID would be expected to be higher than in patients with milder ID [45–47]. In our cohort, 47 patients were enrolled because of MCA and they did not show ID. Indeed pooling data from patients with different ID degree and without ID, we conclude that more severe ID is not statistically related to pathogenic CNVs [4, 5, 29]. Among the consistent number of clinical and history data analyzed, positive family history for ID/MCA/ASD and isolated ASD were found to be associated to pathological aCGH results. We would underline that other congenital anomalies as ocular dysmorphisms (*p*=0.062), hearing loss (*p*=0.127), neurological signs (*p*=0.103), cutaneous dyscromia (*p*=0.08) and endocrinological system involvement (*p*=0.128) are potentially predictors of pathological CNVs.

In this cohort, 37 patients were affected by ASD. The overall diagnostic yield of aCGH for patients with ASD ranges from 18.2–22 % [45, 48–50]. In our cohort the diagnostic yield is consistently greater (around 44 %, 16 patients out 37). Among ASD patients of this case-study, pathogenic CNVs are mostly located at chromosome 1, 4, 6, 8, 21 and 22, that partially confirm the previous results from the literature [45, 51]. In the present study we found a low frequency of abnormal FRAX-A test results as previously described [19].

Some hesitations in using aCGH in clinical setting diagnostic test derive from the difficulties in the efficient discrimination between benign, VOUS and pathogenic CNVs [2, 11, 52]. CNVs can be interpreted as abnormal (pathological CNVs), VOUS and benign. We interpreted CNVs as pathogenic when contained: critical regions of microdeletion/microduplication known syndromes, genes associated with autosomal dominant inherited diseases and when cases with similar phenotypes and overlapping CNVs have already reported. CNVs are likely to be benign if they are reported in controls databases (similar CNVs in at least three healthy individuals in the same “sense”, with an overlap of more than the 50 % and the not-overlapped part less than 100 Kb), if they do not contain genes and/or known regulatory elements. Comparative analysis, with data listed in available large datasets, guide toward the clarification of CNVs clinical impact and interpretation. Multiple sources were considered as level of documentation. All the identified CNVs have been compared to those listed in: the Database of Genomic Variants (DGV, http://projects.tcag.ca/variation) that includes healthy individuals, the pathogenic CNVs databases for patients with ID, ASD and MCA: as the International Standard Cytogenomic Array Consortium Databases (ISCA, https://www.clinicalgenome.or), as well as the Database of Chromosomal Imbalance and Phenotype in Humans using Ensemble Resources (DECIPHER, https://decipher.sanger.ac.uk/). The Database of Genomic Structural Variation (dbVar, http://www.ncbi.nlm.nih.gov/dbvar) including structural variation from both normal control population and disease population has been consulted as well. The genes, involved in the chromosomal region of interest, and their functions have been checked by UCSC Genome Browser (http:// http://genome-euro.ucsc.edu/cgi-bin/hgGateway) and Ensamble Genome Browser (http://www.ensembl.org/index.html). In the interpretative process, each gene, within the CNV as well as neighboring genes, was studied for its potential role in neurological development, by all the available evidence along sources as OMIM, Genereviews, PubMed. The CNVs not associated with previously reported pathogenicity or benignity criteria were estimated as VOUS. The potential pathogenicity of VOUS is reported to be determined by many factors: the “sense” of the rearrangement (deletion or duplication, as the penetrance of duplications is considered lower than of deletions), the size (pathogenic imbalances tend to be larger than benign) and the gene content [7]. We detected and analyzed 25 de novo VOUS and evaluated some cytogenetic indicators: overall size, gain vs loss, presence of multiple rearrangements (complex rearrangements involving several CNVs) and gene content. Only gene content had a significant correlation with ID. The gene content should be evaluated in order to speculate the pathogenicity of VOUS [7, 10, 53].

Due to presence of VOUS, incomplete penetrance, and variable expressivity of CNVs the role of genetic counseling in aCGH testing and CNVs interpretation complements the diagnostic testing. Moreover pre-test counseling cannot be underestimated and should review potential benefits and limitations of the test.

## Conclusion

The achievement of a specific genetic diagnosis improves medical care and allows an accurate recurrent risk counselling for the family. ACGH enables: discovering emerging new syndromes and variable presentations of already characterized ones, deciphering the genetic bases of many syndromes by discovering candidate genes. The current study highlights that a checklist of clinical features for preselecting cases for aCGH analysis with high sensitivity and specificity is difficult to attain. The positive family history for ID/MCA/ASD and the presence of ASD seem to be independent additional clues positively associated to causative CNVs. Among other criteria, ID (with no correlation with the level), eyes anomalies, hearing loss and other neurological signs, cutaneous dyscromia, endocrinological system involvement were also deemed as potentially predictive factor of pathogenic CNVs. VOUS involving gene-rich regions are more frequently associated to ID and pathological phenotypes.

## What’s Known on This Subject

Array-CGH has been defined the first line diagnostic tool in patients sharing intellectual disability and multiple congenital abnormalities. The diagnostic yield is still low and interpretative issues of the results remain elusive.

## What This Study Adds

Phenotypic clues predictive of pathological results have been defined to help in patients’ selection to be studied with array-CGH. Cytogenetic features of rearrangements to be taken into account are provided; variant of uncertain significance showed higher density in patients sharing intellectula disability. A revised flow-chart for patients with intellectual disability is depicted.

### Ethics

This retrospective-prospective study was approved by the Ethics Committee of Federico II University (reference number 5416), and was performed according to the Italian regulations on privacy protection and Helsinki Doctrine for Human Experimentation.

## Additional file

Additional file 1: Supplementary materials. (DOC 36 kb)

## Abbreviations

(aCGH): array-CGH
ABR: brainstem auditory evoked potentials
ASD: autism spectrum disorder
CNVs: copy number variation
EEG: electroencephalogram
ID: intellectual disability
IEMs: inborn errors of metabolism
MCA: multiple congenital anomalies
MRI: Magnetic Resonance Imaging
VOUS: variant of uncertain clinical significance
