# Supplementary material for: New insights in the interpretation of array-CGH: autism spectrum disorder and positive family history for intellectual disability predict the detection of pathogenic variants
Source: Ital J Pediatr. 2016 Apr 12;42:39. doi: 10.1186/s13052-016-0246-7 (PMC4830019; doi:10.1186/s13052-016-0246-7)
Supplement: Supplementary file 1 — Supplementary materials. (DOC 36 kb) [file 13052_2016_246_MOESM1_ESM.doc]

# Supplementary materials

**Clinical-Anamnestic data**

A detailed family history with particular attention to the presence of ID, psychiatric disorders, congenital anomalies and poliabortivity and personal past and recent medical history (including prenatal and birth issues like: IUGR, single umbilical artery, neonatal asphyxia, prematurity) data were collected for each patient. Moreover all the subjects received an accurate clinical and diagnostic evaluation. In detail the physical evaluation included a collection of: auxological parameters (short stature, tall stature, abnormal weight/length for age, macrocephaly and microcephaly), face phenotypic abnormalities (asymmetry, coarse face, small/narrow/elongated/broad/round-shaped/square-shaped/triangular-shaped/flat face, hypotonic, midface hypoplasia), forehead and eyebrows phenotypic abnormalities (frontal bossing, biparietal narrowing, prominent, metopic ridge, high/low anterior hairline, synophrys, arched/sparse/hypoplastic/absent eyebrows), eyes, palpebral fissures and eyelashes phenotypic abnormalities (strabismus, microphthalmos, deeply set eyes, ptosis, short/mongolid and antimongoloid palpebral fissures, telecanthi, epicanthi, hypo/hypertelorism, prominent eyelashes), ears phenotypic abnormalities (aplasia/microtia, large ears, asymmetry, dysplastic ears, simple ear, low-set/posteriorly rotated ears, abnormally modeled helices, overfolded/no fold helices, darwin ‘‘lump’’, ears pits/tags), nose and phyltrum phenotypic abnormalities (choanal atresia/stenosis, cleft nose, large flat/small/short/pinched nose, septum deviation, alae nasi hypoplasia, flat/prominent/high nasal bridge, road/flat/bulbous nasal tip, long/short/smooth/prominent/deep philtrum), oral region, teeth and tongue phenotypic abnormalities (microstomia, macrostomia, mouth asymmetry, open mouth appearance, cleft lip, thin/full/thick/tight lip, cupid bow, hypertrophy of alveolar ridges, cleft/high/narrow/short/wide palate, aplasia/hypoplasia/cleft/large/lobulated/smooth tongue, protruding tongue, macrodontia, microdontia, supernumerary teeth, oligodontia, single central incisor, malocclusion, pierre robin sequence, bifid uvula, widely spaced, abnormally shaped teeth, retro-/micrognathia), neck and thorax phenotypic abnormalities (webbing/short/long neck, loose skinfolds, asymmetry, short/long/flat thorax, pectus carinatum, pectus excavatum, supernumerary/wide-spaced/inverted nipples, pterigium colli, ribs agenesis), joint anomalies (hypo/hypermobilitymobility small joints, dislocations), upper limbs, lower limbs, hands and feet phenotypic abnormalities (ectrodactyly, syndactyly, trident hand, broad thumb, simian crease, bridging crease, absent crease, polydactyly, arachnodactyly, reduction deformity/deficiency, short fingers, brachydactyly, camptodactyly, clinodactyly, syndactyly, tapering finger, fetal pads, sandal gap, clubfoot, pes cavus, varum/valgum knee, lower limbs asymmetry), skin anomalies (hypo/hyperpigmentation of skin, cafe au lait, hemangioma), nails and hair (nails hypoplasia, generalized body hair, fine hair, aplasia cutis, hair hypopigmentation), anus atresia/ stenosis/abnormal position, hearing loss, cardiac malformations (atrial septal defects, ventricular septal defects, outflow tract abnormalities, tetralogy of fallot, hypoplastic left heart syndrome, coarctation of the aorta), gastrointestinal and abdomen malformations (megacolon, duodenal/esophageal stenosis, polysplenia), umbelical and inguinal hernia, CNS malformations (cerebral atrophy, dysmyelination, migration defects, hypoplasy/agenesis of corpus callosum, cerebellar malformations,brainstem hypoplasy, lateral ventricular asymmetry, hypothalamus and pituitary gland lesions), neurological signs (epilepsy, paresis, cerebellar signs), pulmonary malformations (cystic adenomatoid malformation, pulmonary hypoplasy), kidney and urinary tract anomalies (renal hypoplasia/agenesy, double district uretere, vesicoureteral reflux, renal pielectasy, renal ectopia, kidney cysts, renal lithiasis), abnormal external genitalia (cryptorchidism, hypospadias, hypertrophy of labia minora, penoscrotal transposition), ocular malformation (coloboma, chorioretinal malformation, myopia, hypermetropyia, nystagmus, cataract, megalocornea), vertebral anomalies (scoliosis, kyphosis, lordosis, hemispondilus, vertebral agenesis, torticollis, fused vertebra), skeletal dysplasia (acromelia, mesomelia, rizomelia, exostoses, congenital hip dysplasia), immunodeficiency and hematological problems (piastrinopenia, leukemia), endocrinological anomalies (hypothyroidism, precocius puberty, GH deficit, diabetes, adrenal insufficiency).

A neuropsychological assessment and an IQ evaluation were obtained by standardized individually administered tests (Griffiths Mental Development Scales - Extended Revised (GMDS-R) and Wechsler Intelligence Scale for Children (WISC)). ID is sub-grouped in different degree of severity: mild, moderate or severe, depending on the intelligence quotient (IQ): respectively 50 –70, 35-50 and lower than 50. In case of suspect of ASD the Autism Diagnostic Interview-Revised (ADI-R) and Autism Diagnostic Observation Schedule (ADOS) tests were used.

# Cytogenetic analysis methods and CNV evaluation procedure

For aCGH, peripheral blood from the patients was cultured for 72 h in the presence of phytohaemagglutinin. Metaphase spread preparations and GTG-banding (550 bands) were performed according to standard methods. A total of 16 metaphase cells for every sample were analysed. Karyotypes were described according to the International System for Human Cytogenetic Nomenclature and resulted normal. Total genomic patients’ DNA was prepared from ethylenediaminetetraacetic acid (EDTA) blood samples using MasterPure™ DNA Purification Kit for Blood (Epicentre Biotechnologies, Madison, WI, USA). A genome-wide copy number scan was performed using either Constitutional Chip® 4.0 BAC Array (PerkinElmer, Waltham, Massachusetts, USA) with a resolution of 500 Kb in the targeted regions and 2 Mb in the backbone or CytoChip Oligo ISCA 4x44K Array (BlueGnome, Fulbourn, Cambridge, UK) with an average resolution of 75 Kb or NimbleGen CGX-6 Array (Roche, Indianapolis, IN, USA) with a resolution of 50 Kb in the targeted regions and 175 Kb in the backbone, according to the manufacturer’s instructions. Array data were analysed using the OneClickCGH software (Infoquant, London, UK), BlueFuse Multi software (BlueGenome, Fulbourn, Cambridge, UK) and Genoglyphix software (Signature Genomics, Spokane, WA, USA), respectively. The Assemblies Hg18 was initially used being replaced by Hg19 then. Pathogenic or potentially pathogenic CNVs identiﬁed by array CGH were veriﬁed by FISH.

All the identified CNVs have been compared to those listed in: the Database of Genomic Variants (DGV, http://projects.tcag.ca/variation) that includes healthy individuals, the pathogenic CNVs databases for patients with ID, ASD and MCA: as the International Standard Cytogenomic Array Consortium Databases (ISCA, https://www.iscaconsortium.org/index.php/search), as well as the Database of Chromosomal Imbalance and Phenotype in Humans using Ensemble Resources (DECIPHER, https://decipher.sanger.ac.uk/). The Database of Genomic Structural Variation (dbVar, http://www.ncbi.nlm.nih.gov/dbvar) which includes structural variations from both normal control population and disease population has been consulted as well. Moreover the interpretative process has been influenced by specific parental results of aCGH testing.
